# Quantum–mechanical property prediction of solvated drug molecules: what have we learned from a decade of SAMPL blind prediction challenges?

**DOI:** 10.1007/s10822-020-00347-5

**Published:** 2020-10-20

**Authors:** Nicolas Tielker, Lukas Eberlein, Gerhard Hessler, K. Friedemann Schmidt, Stefan Güssregen, Stefan M. Kast

**Affiliations:** 1 Physikalische Chemie III, Technische Universität Dortmund, Otto-Hahn-Str. 4a, 44227 Dortmund, Germany; 2 R&D Integrated Drug Discovery, Sanofi-Aventis Deutschland GmbH, 65926 Frankfurt am Main, Germany; 3 R&D Preclinical Safety, Sanofi-Aventis Deutschland GmbH, 65926 Frankfurt am Main, Germany

**Keywords:** Drug discovery, SAMPL, Solvation model, Quantum chemistry, Integral equation theory, EC-RISM

## Abstract

Joint academic–industrial projects supporting drug discovery are frequently pursued to deploy and benchmark cutting-edge methodical developments from academia in a real-world industrial environment at different scales. The dimensionality of tasks ranges from small molecule physicochemical property assessment over protein–ligand interaction up to statistical analyses of biological data. This way, method development and usability both benefit from insights gained at both ends, when predictiveness and readiness of novel approaches are confirmed, but the pharmaceutical drug makers get early access to novel tools for the quality of drug products and benefit of patients. Quantum–mechanical and simulation methods particularly fall into this group of methods, as they require skills and expense in their development but also significant resources in their application, thus are comparatively slowly dripping into the realm of industrial use. Nevertheless, these physics-based methods are becoming more and more useful. Starting with a general overview of these and in particular quantum–mechanical methods for drug discovery we review a decade-long and ongoing collaboration between Sanofi and the Kast group focused on the application of the embedded cluster reference interaction site model (EC-RISM), a solvation model for quantum chemistry, to study small molecule chemistry in the context of joint participation in several SAMPL (Statistical Assessment of Modeling of Proteins and Ligands) blind prediction challenges. Starting with early application to tautomer equilibria in water (SAMPL2) the methodology was further developed to allow for challenge contributions related to predictions of distribution coefficients (SAMPL5) and acidity constants (SAMPL6) over the years. Particular emphasis is put on a frequently overlooked aspect of measuring the quality of models, namely the retrospective analysis of earlier datasets and predictions in light of more recent and advanced developments. We therefore demonstrate the performance of the current methodical state of the art as developed and optimized for the SAMPL6 p*K*_a_ and octanol–water log *P* challenges when re-applied to the earlier SAMPL5 cyclohexane-water log *D* and SAMPL2 tautomer equilibria datasets. Systematic improvement is not consistently found throughout despite the similarity of the problem class, i.e. protonation reactions and phase distribution. Hence, it is possible to learn about hidden bias in model assessment, as results derived from more elaborate methods do not necessarily improve quantitative agreement. This indicates the role of chance or coincidence for model development on the one hand which allows for the identification of systematic error and opportunities toward improvement and reveals possible sources of experimental uncertainty on the other. These insights are particularly useful for further academia–industry collaborations, as both partners are then enabled to optimize both the computational and experimental settings for data generation.

**Electronic supplementary material:**

The online version of this article (10.1007/s10822-020-00347-5) contains supplementary material, which is available to authorized users.

## Introduction

### Physics-based modeling in drug discovery

Drug discovery is a multidimensional optimization journey starting off from early hit molecules with multiple liabilities to a clinical candidate with desired pharmacokinetic/pharmacodynamic (PK/PD) and safety profile, which requires the parallel monitoring of different properties. While retaining the biological activity against protein target(s) of interest, physicochemical properties like solubility and lipophilicity need to fit a target product profile as well as absorption, distribution, metabolism, excretion, and toxicity, ADMET [1] properties. Precise computational prediction of such properties by in silico methods can significantly reduce the cycle time for drug discovery, as demonstrated by growing interest and application of artificial intelligence (AI) methods [2–6]. While AI methods are empirical and require large amounts of data to learn the structure–property relationship, physics-based methods employ established physical knowledge for property prediction. Here, the quality of the predictions resulting from physics-based methods depends on the level of approximations and parameters used to describe the underlying processes or reactions.

Quantum mechanics (QM) based methods aim at building highly detailed models for calculating the electronic structure of a system described at the lowest possible level of approximations. QM methods may apply wave functions (ab initio), density functional theory (DFT) or semiempirical methods, which are all physics-based and thus use fewer approximations and parameters than empirical force field techniques. Thus, they are of practical value for the design of pharmaceutical compounds, particularly in model systems involving reactions, lacking a detailed parametrization. Only QM-based approaches can be used to predict processes that change the topology of the molecule as a result of chemical reactivity, which can be helpful for optimizing synthetic routes to drug-like compounds. Recently, this has been used at Sanofi and elsewhere in the area of late-stage-functionalization (LSF) [7–9]. Chemical reactivity is also important for the metabolic degradation of pharmaceutical compounds by enzymes of the Cytochrome P450 (CYP) family. Therefore, QM descriptions have been employed in various site of metabolism predictions of drug-like compounds [10, 11].

However, in the early phases of drug discovery during hit finding and early lead optimization, the use of high-quality QM methods is still limited due to resource requirements. Here, many candidate structures need to be evaluated while at the same time the systems to be studied are often very large, such as protein–ligand complexes in structure-based design. The handling of many small molecules is trivially parallel and could therefore be treated with corresponding computing resources available today. Thus, in the early days of computer-aided drug design more than 20 years ago, only simple QM-derived properties such as the molecular electrostatic potential, MEP [12] or torsional energy profiles [13] have been used in industry. The treatment of protein–ligand complexes, however, still constitutes a big challenge for QM-based computational chemistry. On the other hand, force field-based methods that use simple physics models for bonded and non-bonded interactions are well established for investigating conformational properties of proteins and drug-like compounds [14, 15] in drug discovery. They are fast, easy to apply and provide results in a time frame that fits neatly to the requirements of design-make-test-analyze cycles used by project teams throughout industry. This speed advantage comes at the inherent cost to all force field methods that the prediction quality is limited by the chemical space used for parametrization. While high-quality force fields have been developed that are dedicated to proteins, nucleic acids or lipids, general force fields for drug-like small molecules do not reach that same level of accuracy [16]. A compromise towards higher accuracy of force fields consists in augmenting them by torsional potentials customized towards the system under investigation and derived from QM calculations. As an example, for the OPLS3e force field that is used frequently in industry, this process is automated within the Maestro software [17]. This contributed to improvements in free-energy perturbation methods that together with a significant increase in compute power on graphical processing units led to an increased interest in those methods recently [18–21].

Furthermore, supervised machine learning approaches (e.g. 2D/3D-QSAR, quantitative structure–activity relationships) are often used to compensate for limitations in force field-based methods by making use of larger or project-specific datasets. In this situation, quantum mechanics-derived molecular descriptors offer an interesting opportunity to introduce a more physics-based description of molecules into the property prediction. At Sanofi and other pharmaceutical companies semiempirical quantum mechanics approaches like AM1 have been employed to derive molecular orbital (MO-) based descriptors for use in 2D-QSAR [22], but also 3D-QSAR, where the use of MO-based descriptors enabled a more detailed analysis of the factors that affect the affinity of ligands [23].

Deepening the understanding of non-bonding interactions is of great interest for improvements in structure-based design. High-level MP2 (Møller–Plesset 2nd order) and DFT calculations have been employed for studying sigma-hole [24] and π-stacking interactions [25] between aromatic ring systems. Because of the computational expense associated with those methods the size of the systems investigated had to be reduced to a minimal size of two ring systems. With recent advances in improving speed and accuracy of further approximated QM approaches, successful predictions of protein–ligand interactions have been demonstrated using the FMO-DFTB [26] and PM6-D3H4 [27] methods.

Combination of QM methods with AI offers an interesting opportunity to reduce time requirements of QM-based methods. With some investment in computing time, synthetic training sets can be generated in silico directly from QM-based methods. Based on such datasets, progress in the area of deep learning enabled the development of neural network potentials such ANI-1 or ANI-1cxx [28, 29], which give access to coupled cluster-level energies and geometries for large system sizes that are highly relevant to industrial applications. Very recently, limitations in the applicability of ANI molecular potential have also been overcome by extending the training set to include sulfur and halogens [30], which has the advantage that the trained neural network can predict high-level conformational energies or other properties like dipole moments [31] for large molecule sets very quickly. On the other hand, this approach requires a new neural network training cycle for any additional property. Alternative approaches like SchNOrb overcome this obstacle by employing deep learning directly on a ground state wave function from which different properties can be derived [32].

In later stages of lead optimization until early development fewer molecules are subject of more thorough investigations. Thus, more expensive computational studies can be afforded for advanced molecules, prior or subsequent to experimental studies. As an example, physicochemical properties of substances strongly depend on solvent effects, which can be treated explicitly or implicitly by QM methods. This way, the rates of formation of reactive or particularly genotoxic impurities can be estimated by computation. Furthermore, QM methods allow for the prediction of spectral properties of drug candidates, such as chromophores or UV/visible light absorption. Both thermal stability and light-induced decay are important drug product properties to monitor, which determine the drug’s shelf-life and may complicate drug production, logistics and distribution by the need of light protected storage. Beyond that, UV-sensitive drug candidates can give rise to light-induced adverse events summarized as phototoxicity or photoallergy and may require drug labelling or even withdrawal of the drug candidate. In current drug development practice, photosafety testing remains to be an important component for synthetic molecules. While current regulatory guidelines leave flexibility to both scheduling and methods applied, timely prediction and optimization of photosafety is facilitated by QM methods. In the group of the authors, hybrid methods have been pioneered, embedding machine learning and time-dependent TD-DFT calculations to determine UV/vis spectral absorption descriptors of drug candidates in solution. Beyond property prediction, this method was shown effective for the detection of fragmental contributions to toxicity, a key prerequisite for visualization and helpful for guiding drug optimization [33].

Another important aspect in drug development concerns investigations into which molecular species are present in solution and their contributions to bioactivity. This means that properties like purity and physicochemical properties such as log *P*, log *D*, and p*K*_a_ among others will have to be determined with high quality, including clarity about the prevalence of different tautomeric species, where necessary. Here, high-level computational techniques offer additional insights to experimental approaches. For instance, this is done at Sanofi in cases where drug molecules can exist in different isomeric forms in solution. Solvation models coupled to QM calculations such as the EC-RISM approach described below are performed on an ensemble of conformations to provide insights about different isomers, which might influence the biological activity of the molecules. Often, it could be shown that the isomers observed experimentally are significantly more stable compared to other isomers, contributing to a total population of more than 99.9%.

The industrial application of computational methods in drug discovery projects requires high-quality predictions that are validated and generally accepted in the scientific community in order to provide answers accepted by regulatory authorities on one hand and to meet the demand of reducing experimental effort substantially on the other. Therefore, measuring and assessing the predictive quality of the tools is of utmost importance. It is of equal importance, however, that consistent and high-quality data, as typically obtained and archived in industry, are used for such benchmarking purposes. This emphasizes the importance of an exchange of pre-competitive data between industry and academia for method development and for measuring and assessing the predictive quality of the tools.

### Background of SAMPL blind prediction challenges

In this context the SAMPL (Statistical Assessment of Modeling of Proteins and Ligands) blind prediction challenges [34] represent a widely known platform for testing models on high-quality experimental data that are revealed to participants only after they have submitted their predictions. The SAMPL challenges were originally invented and organized by scientists from Stanford University and the software company OpenEye, focusing initially on small molecule solvation free energies (SAMPL0 [35] and SAMPL1 [36]) and later expanding the scope toward tautomerization free energies in water (SAMPL2 [37]) and host–guest binding affinities (SAMPL3 [38] and SAMPL4 [39]). However, solvation free energy challenges remained a central topic throughout running in parallel [37, 38, 40]. Protein–ligand binding pose and affinity predictions were spun-off from SAMPL in the form of “Grand Challenges” organized by the Drug Design Data Resource (D3R) [41] while SAMPL5 was devoted to host–guest binding on the one hand [42] and—as an extraordinarily more complicated problem compared to earlier small molecule SAMPL challenges—distribution coefficients between water and cyclohexane on the other [43]. This particular challenge pushed the computational chemistry community to its limits as it turned out that prediction metrics were substantially worse compared to “simpler” hydration free energy problems. This is related on one hand to the fact that cyclohexane represents a noncommon apolar solvent for pharmaceutical applications. On the other hand, the problem is far more challenging as distribution coefficients (log *D*) at a certain pH imply not only neutral-state partitioning thermodynamics between phases (measured by the log *P*) but also protonation equilibria for tautomerizable compounds. Besides the host–guest problem addressed again during SAMPL6 [44] this subsequent challenge eased the complexity somewhat compared to SAMPL5 as participants were asked to predict aqueous p*K*_a_ values of small molecules [45] and, during SAMPL6 part II, octanol–water log *P* for a selected subset of neutral-state SAMPL6 compounds [46]. As the SAMPL initiative recently received NIH funding, project leader D. L. Mobley and colleagues J. D. Chodera, B. C. Gibb, and L. Isaacs were able to continue on the roadmap, with the SAMPL7 challenge on host–guest and physical property predictions currently running.

Our interest in the SAMPL series of challenges arose from the ongoing developments in the Kast group in the first decade of the twenty-first century in the area of integral equation theories of the liquid state, most prominently in the form of the three-dimensional reference interaction site model (3D RISM) [47–49]. This methodology allows for the approximate calculation of solvation free energies directly from solute–solvent site distribution functions derived from pair interactions and a precomputed pure-solvent “response function” (susceptibility or site density–density correlation function). Particularly important is the possibility to compute—in contrast to computationally more demanding molecular simulations—the solvation free energy analytically, though at the price of added uncertainty due to the so-called closure approximations. Based on a variational analysis of the underlying mathematical concepts it was possible to derive which type of closure approximations satisfy certain conditions of thermodynamic consistency [50] from which a new class of closure approximations could be deduced in 2008, the “*n*^th^ order partial series expansion” (PSE-*n*) [51] which combines numerical stability with satisfactory (though still limited in absolute terms, see below) predictions of solvation free energies compared to reference calculations employing the “hypernetted chain” (HNC) closure. Together with an efficient formulation of the free energy problem suitable for large-scale 3D RISM calculations [52] it is now possible to routinely compute solute–solvent distribution functions and thermodynamic quantities even for very large solutes in various solvents. In the same year 2008, we developed an extension by coupling 3D RISM theory to quantum-chemical calculations for a solvated molecule as a numerically simpler alternative to established coupling schemes [49]. It was termed “embedded cluster reference interaction site model” (EC-RISM) [53] as the solvent impact on the solute’s wave function is modelled by discretizing the solvent charge density from 3D RISM theory to form a set of embedding point charges. From self-consistent calculations of solvent and electronic structure we can compute the solvent-polarized electronic energy and the excess chemical potential, the sum of which represents the free energy of a compound in solution.

One of the first applications of EC-RISM theory was devoted to protonation equilibria, namely the calculation of relative p*K*_a_ differences between similar small molecules for which the approximation artefacts of 3D RISM were expected to largely cancel [53]. At the time of these developments, 2008–2009, the Kast group got into contact with the company Sanofi that recognized the potential of 3D RISM/EC-RISM for pharmaceutical research, which formed the nucleus of a decade-long academia–industry collaboration that is still ongoing. While absolute solvation free energies from RISM calculations were out of reach at that time, the SAMPL2 tautomer challenge was perfectly timed to rigorously assess the quality of the EC-RISM approach, as the methodology—again under the assumption of error cancellation for similar molecular tautomeric states—could demonstrate its potential for application to an important problem occurring during a drug discovery campaign. Tautomers are highly relevant as their state strongly affects the binding of a ligand to a drug target; predicting and controlling tautomer preferences is therefore an important design goal. QM is necessarily an essential modeling component for a microscopic, physics-based approach since chemical reactions are involved.

Hence, we joined forces by exchanging tools and methods and developed a workflow that is still the basis for later challenges to come [54]. Briefly, the experimental SAMPL2 dataset consisted of an “explanatory” (reference data was revealed to the participants) and an “obscure” dataset for which predictions had to be submitted (and another “investigatory” set, for which no experimental numbers were known). The EC-RISM model available at that time was applied to an exhaustively sampled set of conformations, employing a self-consistent point charge approximation for electrostatic solute–solvent interactions. Remarkably, we obtained a root mean square error (RMSE) of 0.57 kcal mol^−1^ for the explanatory set (excluding two highly uncertain compounds) with little procedural optimization, but the performance on the obscure set was disappointing with an RMSE of only 2.91 kcal mol^−1^ (see Table 6 in [54]). Interestingly, the unbalanced chemical diversity of the two datasets could play a role, as the explanatory set consisted mainly of 5-membered rings and the obscure set of 6-membered rings. The reason for the discrepancy remained elusive, yet the overall performance with an average RMSE of 1.93 kcal mol^−1^ was a decent success also in comparison with other participants [55], although this is a rather meaningless finding given the apparent wide distribution of prediction errors.

The situation concerning absolute RISM-based free energy predictions changed when it was recognized roughly 5 years ago that the error is quantitatively related to the partial molar volume (PMV) and the net charge of a solvated molecule [56–59]. This stimulated our interest in combining this idea with the EC-RISM approach to arrive at a quantitative, predictive model for computing chemical potentials in solution. Again, the coincidence with a SAMPL challenge, this time SAMPL5 on cyclohexane–water log *D* at pH 7.4, triggered implementation of such a corrective scheme and application in another joint academic–industrial collaboration to the dataset [60]. In 2015 we were able to calculate hydration free energies with satisfactory accuracy, whereas a p*K*_a_ model, which would require an additional layer of parametrization to account for the thermodynamics of the solvated proton, was not yet finished at the submission deadline. We therefore decided to optimize the transfer free energy prediction between cyclohexane and water by training individual solvation free energy models with only a few adjustable parameters, supplemented with an empirical estimate of most relevant tautomers and associated p*K*_a_ values by the software MoKa [61]. In the absence of disclosed training data related to the real problem, the community was faced with an extremely hard challenge, and the results were accordingly disenchanting [43]. In our case (see Tables 1, 3 in [60]), RMSE values for the MNSOL [62–65] reference dataset in water (including ions) and cyclohexane reached for the best models 2.43 (1.52 excluding ions) and 0.76 kcal mol^−1^, respectively. With the MoKa p*K*_a_ model our SAMPL5 log *D* estimates deviated by as much as 4.61 p*K* unites RMSE (furthermore restricted to so-called batches 0 and 1 of the dataset, leaving out the conformationally more demanding batch 2 due to time constraints), while a crude approximation, namely ignoring the protonation equilibria altogether and estimating log *D* by log *P*, yielded a surprisingly much better RMSE of 2.86 p*K* units.

Better p*K*_a_ predictions are therefore key to improvement. Only after the submission were we able to develop such a linear EC-RISM-based model which requires two adjustable parameters, one for scaling the Gibbs free energy difference between protonated and deprotonated form, and one intercept parameter representing the free proton contribution [60, 66]. Trained on a reference p*K*_a_ database [67] we obtained an overall RMSE including acids and bases of 1.52 p*K* units (Table 2 in [60]), which, applied again to batches 0 and 1 of the total dataset, improved log *D* predictions down to an RMSE of 2.25 p*K* units. Notably, this result is massively influenced by a few drastically deviating outliers that will be further discussed below.

Participating again as joint team in the p*K*_a_ prediction challenge within SAMPL6 in 2017/2018 on small kinase inhibitor fragments was then the logical next step. Here the main difficulty was the presence of multiple protonation sites and the resulting large number of tautomers (or “microstates”), accompanied by several ionization states that are not a priori easy to assign to specific molecular transitions. Participants were also asked—in an investigatory manner—to calculate populations of individual microstates as a function of pH. While the SAMPL5 post-submission p*K*_a_ model was still based on self-consistent atomic site charges for determining the electrostatic contribution to the solute–solvent interactions, we now turned to an EC-RISM variant that allows for using the electrostatic potential arising from the solute’s wave function directly, i.e. formally in an exact manner. This strategy was developed earlier in the context of EC-RISM-type calculations for polarizable solute force fields [68] and was trained again on MNSOL compounds for hydration Gibbs energies and on the reference p*K*_a_ database as used during SAMPL5, yielding a training RMSE of 1.00 p*K* units for the best p*K*_a_ model (see Table 2 in [69]) and 2.20 kcal mol^−1^ for the corresponding hydration free energy model (Table 1 in [69]). Applying this setup to the SAMPL6 dataset turned out to be problematic as not all compounds could be calculated consistently using the exact electrostatic potential, requiring a point charge fallback in certain cases. Consequently, the prediction RMSE suffered, reaching 1.70 p*K* units for the model determined as optimal during training.

After the challenge ended we detected the source of the convergence problem for selected compounds, an inadequate consideration of the aperiodic electrostatic potential under otherwise periodic boundary conditions used within 3D RISM [69]. Correcting for these artefacts facilitated consistent exact potential calculations for the entire dataset, resulting in RMSEs of 2.04 kcal mol^−1^, 1.04 and 1.13 (two conformations)/1.15 (single best conformation) p*K* units for hydration free energies, p*K*_a_ reference set, and SAMPL6 test set, respectively.

Finally, SAMPL6 part II provided participants with the additional opportunity to predict neutral-state transfer free energies, i.e. octanol–water log *P*. Without changing the water setup compared to the optimal post-submission SAMPL6 approach we only optimized a solvation free energy model for octanol, trained again against MNSOL reference data, that requires two parameters and that reflects the saturated water content of the octanol phase adequately. This “wet” octanol model was reasonably successful without any post-submission optimization, as it exhibited an RMSE of only 0.47 p*K* units (see Table 3 in [70]), but one has to consider the small dynamic range of experimental values in this case.

### Outline

Given the optimized strategies developed particularly for SAMPL6 it appears to be timely to re-address SAMPL2 and SAMPL5 datasets in order to find out whether progress has been made on all fronts. Moreover, the strategy has been successfully benchmarked against other independent simulation-based approaches for calculating tautomerization equilibria of natural and artificial nucleobases very recently [71], also augmented by an alternative route comprising explicit high-level gas phase calculations combined with hydration Gibbs energies for individual species (unlike the “direct” approach to compute a solute’s Gibbs energy as sum of electronic energy and excess chemical potential as used throughout in SAMPL2, -5, and -6). We therefore turned to the older datasets in order to measure the performance of the SAMPL6 setup and workflow, optimizing and retraining only the cyclohexane model in order to find out possible sources of systematic error. This is then followed by re-analysis of the SAMPL2 tautomer dataset in the same spirit. The—somewhat surprising—results are finally discussed in light of future challenges on both the computational and the experimental side, both domains potentially benefitting from deepened academia–industry collaborations as a perspective.

## Computational details

For re-addressing the SAMPL5 challenge compounds, two setups were applied and compared, the original SAMPL5 methodology [60], here extended by covering the whole compound set including batch 2 which was not possible back then, and an extension of the SAMPL6 models [69, 70] to address cyclohexane solution thermodynamics. For the latter, the “MP2/6-311+G(d,p)/*φ*_opt_” water model and the corresponding two-parameter p*K*_a_ model from the SAMPL6 p*K*_a_ challenge were used unchanged for calculating the Gibbs energy of the molecules in the aqueous phase and the acidity constants [69, 70]. Also for the SAMPL6 setup, we developed new cyclohexane models trained to reproduce solvation Gibbs energies found in the MNSOL dataset [62–65], using the same cyclohexane susceptibility and solution phase training set structures as before for SAMPL5 [60] and, unlike the SAMPL5 workflow where we assumed identical geometries in gas and solution phase, explicit gas phase re-optimized conformers on the B3LYP/6-311+G(d,p) level of theory using Gaussian 09 Rev. A.02 [72]. In contrast to the original SAMPL5 setup where only the most abundant tautomer and corresponding Corina-generated 3D conformations were taken as representative for a given compound, for the SAMPL6 setup *every* tautomer state generated by MoKa [61] with initial 3D conformations taken from Corina [73] was investigated. Enumeration of stereocenters was not necessary as all stereocenters were explicitly defined in the input data and no new stereocenters were produced during tautomerization. As we found during SAMPL6 that accounting for conformational flexibility is relevant [69], we used five SAMPL5 compound structures with lowest PCM energies in the respective solvent for each individual tautomeric state, instead of just the minimum structure within the SAMPL5 setup.

The same workflow used for generating the conformations for batch 0 and batch 1 during the SAMPL5 challenge was repeated here to sample the conformations for batch 2 and the alternative tautomers: first, for each molecule 200 conformations were generated using the EmbedMultipleConfs function of RDKit [74, 75]. These conformations were then pre-optimized using antechamber from the Amber12 software package with an implicit solvent model using the dielectric constants of water and cyclohexane, respectively to account for solvation effects with AM1-BCC charges and GAFF version 1.7 (identical with 1.4) parameters for the non-bonded terms [76–79]. The resulting structures were clustered based on the following criteria: all conformations with a force field energy at least 5 kcal mol^−1^ higher than the lowest energy conformation found were discarded. The minimum structure was then assigned as the first cluster and the structural root mean square difference (RMSD) to the next best structure was determined using the GetBestRMS function of RDKit. If this structure had an RMSD of less than 0.5 Å the structure was assumed to be properly represented by the existing cluster. In case of a larger RMSD the structure was instead assigned as a new cluster to which all further conformations were compared as well. All cluster representatives generated in this way were then optimized quantum-chemically at the IEFPCM/B3LYP/6-311+G(d,p) level of theory using Gaussian 09 Rev. A.02 [72]. Within the SAMPL6 setup, up to five conformations with the lowest PCM (polarizable continuum model) energy were taken from those cluster representatives to calculate the Gibbs energy in solution using EC-RISM and a similar partition function approach as during the SAMPL6 challenge, whereas only the globally optimal conformation (for the MoKa-determined dominant tautomer) was selected for the SAMPL5 setup.

The Gibbs energy *G* of a species immersed in a solvent *i* is defined as the Boltzmann-weighted sum over state-specific electronic energies, *E*^sol^, in solution and (corrected) excess chemical potentials by

1 $$ G(i) =  - RT\ln \sum\limits_{{tc}} \exp \left[ { - \left( {E_{{tc}}^{{{\text{sol}}}} (i) + \mu _{{tc}}^{{{\text{ex}},{\text{corr}}}} (i)} \right)/RT} \right] $$

with molar gas constant *R* and absolute temperature (25 °C) *T*, and where *t* and *c* denote the tautomeric state and the conformer, respectively. The correction to the EC-RISM-derived excess chemical potential is defined by [60, 69, 70]

2 $$\mu _{{tc}}^{{{\text{ex}},{\text{corr}}}}  = c_{\mu } \mu _{{tc}}^{{{\text{ex}}}}  + c_{V} V_{{{\text{m}},tc}}  + c_{q} q + d $$

with adjustable, solvent-specific parameters *c* scaling the RISM chemical excess potential *μ*^ex^, the PMV *V*_m_, and the net charge *q*, and an optional intercept parameter *d* can be employed. The solvation free energy follows by subtracting the gas phase energy, ignoring thermal corrections. For evaluating the log *D* of the SAMPL5 compounds these Gibbs energies in water (W) and cyclohexane (C) enter the partition coefficient log *P* directly via

3 $$ \log \;P = \frac{G(W) - G(C)}{{RT\ln 10}}. $$

*G* actually corresponds to standard state quantities as calculations were performed at infinite dilution in solvents assumed at 1 bar pressure by definition of the density. The distribution coefficient at pH 7.4 then follows by accounting for the p*K*_a_ (here computed according to the optimized SAMPL6 setup) from

4 $$ \log \;D_{7.4} = \log \;P - \log (1 + 10^{{{\text{p}}K_{{\text{a}}} - 7.4}} ) $$

for bases (if no deprotonation site is detected or if p*K*_b_ < p*K*_a_) and

5 $$ \log \;D_{7.4} = \log \;P - \log (1 + 10^{{{7}{\text{.4}} - {\text{p}}K_{{\text{a}}} }} ) $$

for the acidic compounds. The approach was applied to the three components of the SAMPL5 dataset, batches 0, 1, and 2. Batches 0 and 1 were already treated in our earlier paper [60] within the SAMPL5 setup that was extended here to cover the most abundant tautomer and conformer also for batch 2 species, while the SAMPL6 setup was applied to the complete spectrum of tautomers and corresponding five dominant conformers for all three batches. Note that we detected an error in our original SAMPL5 submission paper [60] where we accidentally applied the base equation to the four acids contained in batch 1 of the dataset, which slightly changes the statistical metrics, to be corrected below.

An important difference between these new SAMPL6-style calculations and the previous SAMPL5 setup concerns the alternative route via explicit solvation free energies substituting *G* in Eq. (3) as given by Eq. (14) in [60]. Physically, this choice makes no difference, as the real gas phase state of the compound is the same when it is dissolved in water or cyclohexane. In the previous calculations we, however, optimized the solvation free energy models for a given set of gas phase and solution state structures that were generated and optimized independently as they arose from separate conformational searches in solution. We accounted for this formal, artificial term by a virtual reorganization energy difference as this approach worked well at that time. Here, we switched to the physically more plausible way of directly calculating the Gibbs energy in solution as done throughout in later challenges.

For consistency with the original SAMPL5 challenge, SAMPL5-style calculations on batch 2 compounds were conducted using the EC-RISM and 3D RISM settings employed during SAMPL5 [60]. For the new SAMPL6-style calculations all of the EC-RISM and 3D RISM settings were chosen identical to those used in the SAMPL6 part II log *P* challenge [70] that slightly differ from SAMPL5 settings, entailing minor numerical differences even when the original cyclohexane model was applied to original SAMPL5 training set structures.

For the SAMPL2 tautomer dataset [54] we followed a similar route as for the direct SAMPL5 Gibbs energy calculations by defining the tautomerization reaction Gibbs energy in water for a state change *a* → *b* as

6 $$ \Delta G^{0} = G_{b} ({\text{W}}) - G_{a} ({\text{W}}), $$

here employing the SAMPL6 water model. We used the same set of exhaustively sampled OH rotamers as before, optimized again on the IEFPCM/B3LYP/6-311+G(d,p) level of theory using Gaussian 09 Rev. D.01 [80] for solution phase structures, consistently with SAMPL6. An alternative route is provided by an explicit thermodynamic cycle as used by us in [71], where we added a high-level [CCSD(T)/cc-pVTZ] gas phase energy difference, calculated using the ORCA [81] software and applying the RI-F12 approximation [82, 83], to the difference between explicit hydration Gibbs energies computed at the SAMPL6 level, including thermal correction computed by vibrational analysis on optimized B3LYP/6–311 + G(d,p) structures using Gaussian 09 Rev. D.01 [80]. For both routes we calculated the free energy per species via a partition function approach averaging over Gibbs energies of all rotamers in solution and—for the indirect route—also in the gas phase to determine the total free energy difference, similar to our recent nucleobase analysis [71].

## Results and discussion

### Cyclohexane training set

In total four different cyclohexane models were newly trained using 1–3 free parameters to fully capture the range of possible corrections for cyclohexane, extending the range of models examined during SAMPL5 [60] and inspired by the insight from the recent SAMPL6 challenges [69, 70]. In particular, the octanol–water challenge [70] showed that a two-parameter model (termed here “2-par”) that scales the excess chemical potential and the PMV performed best, whereas at the time of the SAMPL5 challenge for cyclohexane we tested only models containing the intercept term *d*, scaling either the PMV contribution (termed here “2-par-I” and “(*c*_*μ*_ = 1)” in [60]) or both PMV and excess chemical potential terms (termed here “3-par” and “(*c*_*μ*_ opt)” in [60]). For completeness we also tested a conservative model where only the PMV expression is scaled, termed “1-par”.

**Fig. 1:**
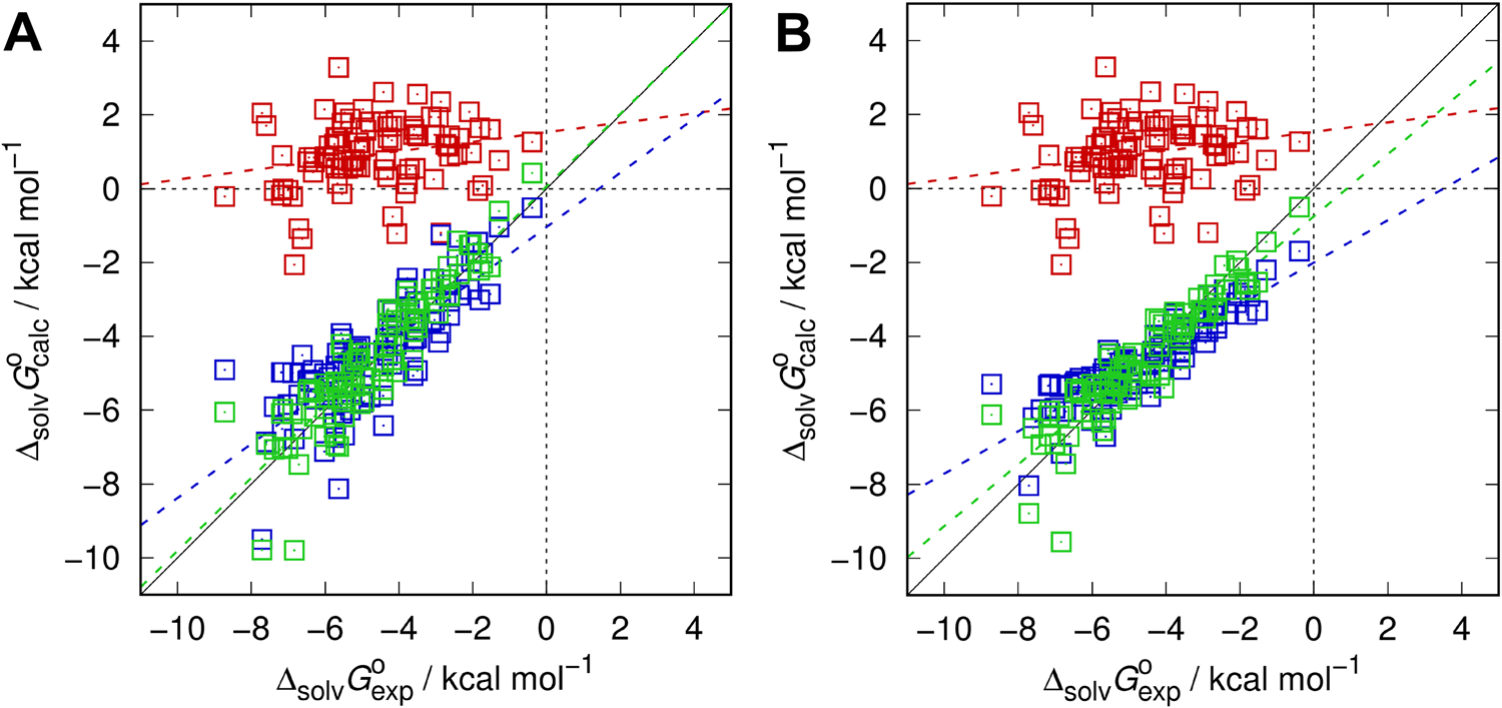
Gibbs energies of solvation in cyclohexane from optimized 3D RISM calculations vs. the experimental results from the MNSOL database. Uncorrected data is shown by red squares in both panels. **A** 1-par (dark blue), 2-par (green) and **B** 3-par (green), 2-par-I (dark blue)

Results for the various models are shown in Table 1 (water model metrics according to the SAMPL6 setup is presented for completeness, see [69]) and Fig. 1. The results obtained here for parameters and statistical metrics are very similar to the original SAMPL5 numbers [60], while the gas phase optimization slightly improves the 3-par model only. A notable difference to the previous paper is reflected by the 2-par model whose development essentially followed the successful octanol model applied during SAMPL6 part II [70]. As expected from the octanol approach, the 2-par model (not tested during SAMPL5) performs very well on the training set, with a root-mean-square error (RMSE) being only slightly worse than the best, but with a slope near one and an intercept near zero accompanied by a near optimal coefficient of determination *R*^2^ indicating a well-balanced approach. One would therefore expect superior performance when applied to the SAMPL5 test set, but in general it is very likely that the improved SAMPL6 water/p*K*_a_ model should exhibit the larger effect on predictions.

**Table 1:** Regression parameters of optimized 3D/EC-RISM/PSE-2-based Gibbs energy of solvation models (*c*_*µ*_, *c*_*V*_/kcal mol^−1^ Å^−3^, *c*_*q*_/kcal mol^−1^ e^−1^, *c*_*d*_/kcal mol^−1^) along with statistical metrics (root-mean-square error RMSE/kcal mol^−1^, mean absolute error MAE/kcal mol^−1^, mean signed error MSE/kcal mol^−1^, slope *m′*, intercept *b′*/kcal mol^−1^, and coefficient of determination *R*^2^ from descriptive regression). Water model data correspond to the “MP2/6-311+G(d,p)/*φ*_opt_” approach in [69]

Solvent	RMSE	MAE	MSE	*m′*	*b′*	*R*^2^	*c*_*µ*_	*c*_*V*_	*c*_*q*_	*c*_*d*_
Water										
All	2.04	1.43	− 0.26	1.00	− 0.35	1.00	–			–
Neutrals	1.56	1.13	− 0.36	0.97	− 0.47	0.89	–	–	–	–
Anions	3.07	2.46	0.01	1.10	7.18	0.94	–	–	–	–
Cations	2.98	2.10	0.02	0.96	− 2.62	0.85	–	–	–	–
Cyclohexane										
Uncorrected	5.86	5.60	5.60	0.13	1.53	0.05	–	–	–	–
1-par	1.07	0.86	0.20	0.73	− 1.04	0.62	–	− 0.14923	–	–
2-par	0.77	0.58	0.11	0.99	0.06	0.83	2.0184	− 0.17795	–	–
2-par-I	0.90	0.73	0.00	0.57	− 2.00	0.76	–	− 0.10894	–	− 1.6593
2-par-I(5)	0.88	0.70	0.00	0.59	− 1.94	0.77	–	− 0.10811	–	− 1.6566
3-par	0.68	0.50	0.00	0.84	− 0.75	0.83	1.8516	− 0.14692	–	− 1.0842
3-par(5)	0.76	0.56	0.00	0.84	− 0.73	0.84	1.8444	− 0.14703	–	− 1.0479

For consistency with the SAMPL6 part II representations *c*_*V*_ corresponds to PMVs computed via the total correlation function route [84, 85] using an experimental isothermal compressibility of 1.1197 × 10^−9^ Pa^−1^ for cyclohexane [86] and the RISM estimate of 0.717062 × 10^−9^ Pa^−1^ for water. “(5)” after the solvent model code indicates SAMPL5 models from [60]. Optimized solution and gas phase structures are provided as Online Resource 1; calculated data, also split into separate components, are provided as Online Resource 2

### SAMPL5 revisited

**Fig. 2:**
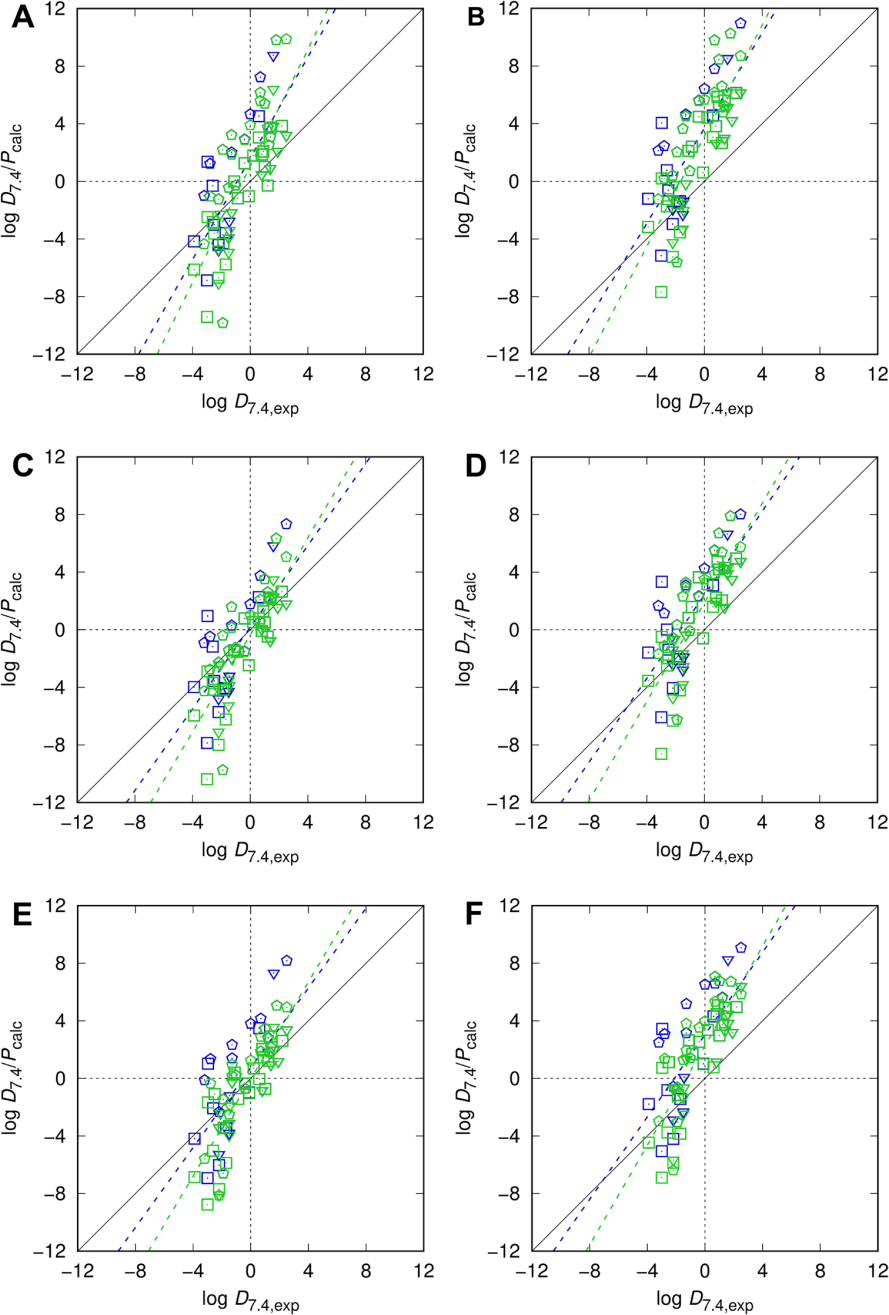
Partition (dark blue) and distribution coefficients (green) calculated using EC-RISM with the SAMPL6- (**A**–**D** 1-par, 2-par, 2-par-I, 3-par) and SAMPL5-type [**E** and **F** 2-par(5), 3-par(5)] models for water and cyclohexane compared with the experimental results (excluding SAMPL5_083, for which MP2 energies could not be obtained). Compounds of batches 0, 1, and 2 are shown as triangles, squares, and pentagons, respectively. Dashed lines indicate descriptive linear regression results

The four new optimized cyclohexane models and the best-performing SAMPL6 water/p*K*_a_ models were then applied to the SAMPL5 dataset, this time to all batches 0, 1, and 2 as batch 2 was left out in our earlier SAMPL5 paper [60], and extended by covering multiple tautomers and conformers. For a complete comparison we also re-applied the original SAMPL5 setup (water and cyclohexane models, point charge electrostatics, globally optimal MoKa-determined tautomer and sampled conformer set) to the full set of batches in order to clarify possibly different trends depending on the choice of compound sets that might bias the analysis. As before, we furthermore show results for both, the pure neutral state partition coefficient, log *P*, and the target quantity, the distribution coefficient at pH 7.4, log *D*_7.4_, collected in Tables 2, 3, 4 and 5 and in Fig. 2.

**Table 2:** Statistical metrics (root-mean-square error RMSE, mean absolute error MAE, mean signed error MSE, and slope *m′*, intercept *b′*, and coefficient of determination *R*^2^ from descriptive regression) for all compounds from SAMPL6-type models for water [MP2/6-311+G(d,p)/PSE-2] and cyclohexane (PSE-2) and the original SAMPL5 setup

Setup	Observable	Cyclohexane mod	Batches	RMSE	MSE	MAE	*R*^2^	*m′*	*b′*
SAMPL6	log *P*	1-par	0 + 1 + 2	3.40	1.25	2.59	0.52	1.76	1.60
		2-par	0 + 1 + 2	4.36	3.38	3.67	0.56	1.65	3.69
		2-par-I	0 + 1 + 2	2.33	− 0.01	1.76	0.54	1.4	0.18
		3-par	0 + 1 + 2	3.18	2.21	2.68	0.57	1.45	2.42
	log *D*_7.4_	1-par	0 + 1 + 2	3.23	0.59	2.50	0.63	2.02	1.07
		2-par	0 + 1 + 2	3.97	2.72	3.40	0.65	1.92	3.15
		2-par-I	0 + 1 + 2	2.46	− 0.67	1.71	0.67	1.69	− 0.35
		3-par	0 + 1 + 2	2.88	1.55	2.44	0.66	1.72	1.89
SAMPL5	log *P*	2-par-I(5)	0 + 1 + 2	2.33	0.55	1.79	0.55	1.39	0.73
		3-par(5)	0 + 1 + 2	3.63	2.85	3.10	0.57	1.43	3.04
	log *D*_7.4_	2-par-I(5)	0 + 1 + 2	2.32	− 0.37	1.76	0.68	1.69	− 0.05
		3-par(5)	0 + 1 + 2	3.11	1.92	2.74	0.66	1.73	2.27

Optimized solution structures are provided as Online Resource 3; calculated data, also split into separate components, as Online Resource 4

**Table 3:** Statistical metrics (root-mean-square error RMSE, mean absolute error MAE, mean signed error MSE, and slope *m′*, intercept *b′*, and coefficient of determination *R*^2^ from descriptive regression) separated by batches using the SAMPL6-type models for water and cyclohexane and the original SAMPL5 setup excluding SAMPL5_083

Setup	Observable	Cyclohexane mod	Batches	RMSE	MSE	MAE	*R*^2^	*m′*	*b′*
SAMPL6	log *P*	1-par	0 + 1	2.29	0.13	1.77	0.63	1.56	0.43
			2	4.74	3.18	4.01	0.54	2.04	3.56
		2-par	0 + 1	3.18	2.37	2.59	0.66	1.52	2.64
			2	5.87	5.14	5.53	0.59	1.82	5.44
		2-par-I	0 + 1	1.99	− 0.65	1.57	0.62	1.31	− 0.49
			2	2.83	1.10	2.09	0.53	1.58	1.31
		3-par	0 + 1	2.44	1.49	1.97	0.63	1.36	1.68
			2	4.15	3.47	3.93	0.60	1.56	3.67
	log *D*_7.4_	1-par	0 + 1	2.45	− 0.59	1.88	0.77	1.89	− 0.12
			2	4.26	2.62	3.58	0.63	2.18	3.05
		2-par	0 + 1	2.88	1.65	2.49	0.74	1.85	2.09
			2	5.36	4.59	4.98	0.66	1.95	4.94
		2-par-I	0 + 1	2.44	− 1.37	1.73	0.74	1.64	− 1.04
			2	2.48	0.55	1.66	0.64	1.71	0.80
		3-par	0 + 1	2.33	0.77	1.91	0.72	1.69	1.13
			2	3.65	2.92	3.38	0.68	1.69	3.17
SAMPL5	log *P*	2-par-I(5)	0 + 1	1.99	− 0.09	1.48	0.61	1.35	0.09
			2	2.83	1.67	2.32	0.52	1.39	1.81
		3-par(5)	0 + 1	2.86	2.08	2.41	0.65	1.41	2.30
			2	3.86	2.98	3.54	0.67	1.81	3.27
	log *D*_7.4_	2-par-I(5)	0 + 1^a^	2.25	− 0.86	1.63	0.71	1.60	− 0.54
			2	2.44	0.48	1.99	0.69	1.81	0.77
		3-par(5)	0 + 1^b^	2.59	1.31	2.29	0.70	1.66	1.67
			2	4.68	4.17	4.29	0.56	1.40	4.32

^a–b^Corrected results for SAMPL5 setup, original values [60] for RMSE, MSE, *R*^2^, *m′*, *b′*:

^a^2.15, − 0.53, 0.59, 1.36, − 0.34

^b^2.76, 1.64, 0.59, 1.42, 1.87

**Table 4:** Experimental distribution coefficients and calculated partition (log *P*) and distribution (log *D*) coefficients for all models of the SAMPL5 challenge, for SAMPL5 [60] and SAMPL6-type setups

SAMPL5 ID	log *D*_7.4,exp_	log *P* 2-par-I(5)	log *P* 3-par(5)	log *P* 1-par-	log *P* 2-par	log *P* 2-par-I	log *P* 3-par	log *D*_7.4_ 2-par-I(5)	log *D*_7.4_ 3-par(5)	log *D*_7.4_ 1-par	log *D*_7.4_ 2-par	log *D*_7.4_ 2-par-I	log *D*_7.4_ 3-par
Batch 0													
003	1.90	1.17	3.19	2.09	4.22	1.54	3.51	1.17	3.19	2.09	4.22	1.54	3.51
015	− 2.20	− 5.28	− 2.87	− 4.76	− 1.92	− 4.79	− 2.41	− 8.08	− 5.67	− 7.07	− 4.23	− 7.10	− 4.72
017	2.50	3.39	6.39	3.20	6.14	1.81	4.75	3.39	6.39	3.20	6.14	1.81	4.75
020	1.60	1.98	3.83	3.83	5.12	2.28	3.91	1.98	3.83	3.83	5.12	2.28	3.91
037	− 1.50	− 3.79	− 2.31	− 3.91	− 2.29	− 4.27	− 2.79	− 3.95	− 2.47	− 4.92	− 3.30	− 5.27	− 3.80
045	− 2.10	− 2.42	− 0.64	− 2.26	− 0.22	− 2.43	− 0.67	− 2.42	− 0.64	− 2.26	− 0.22	− 2.43	− 0.67
055	− 1.50	− 3.13	− 1.31	− 3.91	− 1.53	− 3.50	− 1.65	− 3.13	− 1.31	− 3.91	− 1.53	− 3.50	− 1.65
058	0.80	− 0.83	1.16	0.47	2.64	0.03	2.00	− 0.83	1.16	0.47	2.64	0.03	2.00
059	− 1.30	− 0.25	1.32	− 2.17	− 0.17	− 1.96	− 0.36	− 0.25	1.32	− 2.17	− 0.17	− 1.96	− 0.36
061	− 1.45	− 1.19	0.08	− 2.76	− 1.37	− 3.22	− 1.89	− 1.91	− 0.65	− 3.39	− 2.00	− 3.86	− 2.53
068	1.40	0.95	3.33	0.91	2.99	− 0.76	1.57	0.95	3.33	0.91	2.99	− 0.76	1.57
070	1.60	7.32	8.25	8.76	8.52	5.84	6.65	3.56	4.48	6.40	6.16	3.48	4.29
080	− 2.20	− 3.42	− 0.71	− 4.69	− 1.21	− 4.11	− 1.40	− 3.42	− 0.71	− 4.69	− 1.21	− 4.11	− 1.40
Batch 1													
004	2.20	2.60	4.96	3.85	6.12	2.64	4.96	2.60	4.96	3.84	6.12	2.63	4.95
005	− 0.86	− 1.44	1.68	− 1.17	2.41	− 1.54	1.58	− 1.44	1.68	− 1.18	2.41	− 1.54	1.58
007	1.40	2.91	4.90	3.73	5.59	2.22	4.30	2.91	4.90	3.73	5.59	2.22	4.30
010^a^	− 1.70	− 3.45	− 1.43	− 3.60	− 1.38	− 4.05	− 2.03	− 5.88	− 3.85	− 5.77	− 3.55	− 6.23	− 4.21
011^b^	− 2.96	1.03	3.43	1.36	4.05	0.95	3.34	− 1.67	0.74	− 2.48	0.21	− 2.89	− 0.50
021	1.20	1.22	3.72	− 0.28	2.65	− 0.48	2.04	1.22	3.72	− 0.28	2.65	− 0.48	2.04
026^c^	− 2.60	− 2.08	− 0.82	− 0.31	0.77	− 1.18	0.02	− 5.02	− 3.76	− 2.82	− 1.74	− 3.69	− 2.49
027	− 1.87	− 3.44	− 1.16	− 4.29	− 1.48	− 4.12	− 1.83	− 3.44	− 1.16	− 4.34	− 1.53	− 4.17	− 1.88
042	− 1.10	0.40	2.63	0.01	2.12	− 1.44	0.83	0.40	2.63	0.01	2.12	− 1.44	0.83
044	1.00	− 0.74	2.97	1.00	5.21	0.50	4.19	− 0.74	2.97	1.00	5.21	0.50	4.19
046	0.20	0.70	3.38	1.79	4.42	0.53	3.17	0.70	3.38	1.79	4.42	0.53	3.17
047	− 0.40	− 0.35	2.53	1.26	4.48	0.79	3.64	− 0.35	2.53	1.26	4.48	0.79	3.64
048	0.90	1.47	5.07	2.08	5.86	1.28	4.74	1.47	5.07	2.08	5.86	1.28	4.74
056	− 2.50	− 1.10	1.12	− 3.02	− 0.63	− 3.56	− 1.37	− 1.10	1.12	− 3.63	− 1.24	− 4.17	− 1.98
060^d^	− 3.90	− 4.19	− 1.79	− 4.17	− 1.21	− 3.99	− 1.58	− 6.86	− 4.45	− 6.13	− 3.17	− 5.95	− 3.54
063	− 3.00	− 6.93	− 5.06	− 6.88	− 5.15	− 7.86	− 6.08	− 8.77	− 6.90	− 9.41	− 7.68	− 10.39	− 8.61
071	− 0.10	− 0.99	1.02	− 1.03	0.61	− 2.47	− 0.60	− 1.02	0.99	− 1.04	0.61	− 2.48	− 0.60
072	0.60	3.49	4.30	4.53	4.55	2.27	3.09	− 0.05	0.76	3.04	3.07	0.78	1.60
081	− 2.20	− 6.02	− 4.20	− 4.41	− 2.96	− 5.72	− 4.05	− 7.69	− 5.86	− 6.68	− 5.23	− 7.99	− 6.32
090	0.80	2.04	4.46	1.87	3.82	− 0.08	2.23	2.04	4.46	1.87	3.82	− 0.08	2.23
Batch 2													
002	1.40	2.17	4.35	3.07	5.22	2.06	4.21	2.17	4.35	3.07	5.22	2.06	4.21
006	− 1.02	0.20	1.41	− 0.28	0.71	− 1.26	− 0.09	0.20	1.41	− 0.28	0.71	− 1.26	− 0.09
013	− 1.50	− 2.53	1.28	− 0.44	3.64	− 1.45	2.31	− 2.53	1.28	− 0.44	3.64	− 1.45	2.31
019	1.20	2.81	5.61	3.74	6.59	2.61	5.38	2.77	5.57	3.74	6.59	2.61	5.38
024	1.00	3.46	6.75	5.40	8.43	3.51	6.70	3.46	6.75	5.40	8.43	3.51	6.70
033	1.80	5.06	6.72	9.80	10.24	6.33	7.90	5.06	6.72	9.80	10.24	6.33	7.90
049	1.30	1.80	3.81	2.50	4.79	2.25	4.25	1.80	3.81	2.50	4.79	2.25	4.25
050	− 3.20	− 0.11	2.49	− 1.00	2.12	− 0.91	1.67	− 5.58	− 2.98	− 4.36	− 1.24	− 4.27	− 1.69
065	0.70	1.88	7.06	6.16	9.79	0.54	5.53	1.88	7.06	6.16	9.79	0.54	5.53
067	− 1.30	1.40	3.15	3.23	4.54	1.59	3.26	0.17	1.94	3.23	4.54	1.59	3.26
069	− 1.30	2.34	5.18	2.01	4.64	0.28	3.08	0.95	3.79	1.86	4.49	0.13	2.93
074	− 1.90	− 6.61	− 3.04	− 9.85	− 5.62	− 9.76	− 6.25	− 6.61	− 3.04	− 9.85	− 5.62	− 9.76	− 6.26
075	− 2.80	1.35	3.07	1.22	2.46	− 0.48	1.15	− 0.36	1.37	− 1.05	0.18	− 2.75	− 1.13
082	2.50	8.17	9.06	12.15	10.96	7.34	8.02	4.94	5.84	9.88	8.69	5.06	5.75
083^e^	− 1.90	–	–	–	–	–	–	–	–	–	–	–	–
084	0.00	3.79	6.52	4.66	6.42	1.77	4.25	1.25	3.97	3.90	5.67	1.02	3.50
085	− 2.20	− 2.33	− 0.57	− 1.24	0.39	− 2.29	− 0.56	− 8.14	− 6.39	− 1.24	0.39	− 2.29	− 0.56
086	0.70	4.15	6.59	7.23	7.80	3.74	5.52	2.89	5.32	5.58	6.15	2.09	3.87
088	− 1.90	− 1.46	− 0.62	2.19	2.02	− 0.41	0.35	− 1.46	− 0.62	2.19	2.02	− 0.41	0.35
092	− 0.40	− 0.71	3.52	2.91	5.61	− 1.51	2.33	− 0.71	3.52	2.87	5.56	− 1.55	2.28

^a–d^Corrected results for SAMPL5 setup, original data [60] for log *P*(2-par-I(5), 3-par) and log *D*_7.4_(2-par-I(5), 3-par):

^a^ − 3.45, − 1.43, − 3.46, − 1.43

^b^1.03, 3.43, 1.03, 3.43

^c^ − 2.08, − 0.82, − 2.08, − 0.82

^d^ − 4.19, − 1.79, − 4.19, − 1.79

^e^Excluded as MP2 energies could not be calculated

**Table 5:** Statistical metrics (root-mean-square error RMSE, mean absolute error MAE, mean signed error MSE, and slope *m′*, intercept *b′*, and coefficient of determination *R*^2^ from descriptive regression) for the p*K*_a_ values predicted using the SAMPL5 and SAMPL6 setups compared with the Chemicalize [87] predictions

p*K*_a_ model	RMSE	MSE	MAE	*R*^2^	*m'*	*b'*
SAMPL5	2.07	− 0.57	1.54	0.72	0.88	0.21
SAMPL6	2.10	0.58	1.45	0.73	0.94	0.99

As a first result, predictions for batch 2, which contains mostly larger molecules compared to batches 0 and 1 and which therefore deviates most from PMV correction and p*K*_a_ training set species, are systematically worse than for the other batches. For the SAMPL5 setup the cause could be related the higher likelihood of missing relevant tautomers or conformers, but this tendency is consistently found for both, SAMPL5 and SAMPL6 setups. Given the resulting uncertainty in RMSE metrics, we find—to our surprise—basically no significant difference between the two setups, despite the fact that individual training set results (solvation free energies and p*K*_a_) were markedly improved between SAMPL5 and SAMPL6. This discrepancy between training and test set performance is also reflected by the fact that the expectedly worse 2-par-I model turns out, again as during SAMPL5, to be better than the 3-par approach. And—most strikingly—the well-balanced 2-par model introduced for the SAMPL6 setup, which yielded excellent predictions for octanol–water log *P* during SAMPL6 part II, is even the worst of all models tested in terms of RMSE and mean signed error (MSE). Still, as expected, log *P* correlates worse than predicted log *D* with experiments measured by error metrics and *R*^2^, but regression slopes *m*′ deviate even more strongly from unity by inclusion of p*K*_a_, and models without an explicit intercept parameter (i.e. all except 2-par-I) show a regression intercept *b*′ substantially far off from zero. All these findings indicate a systematic problem that cannot easily be identified as originating from a theoretical or an experimental source. With the present EC-RISM capabilities it appears impossible to obtain RMSEs better than 2–3 for log *D*.

Regression slopes much larger than unity are a signature of systematic asymmetry, as solubilities of highly water-soluble compounds in cyclohexane are strongly underestimated (or their solubility in water overestimated), and vice versa for highly cyclohexane-soluble species. Near the extremes of the dynamic range, we hence observe extraordinarily large log *D* errors exceeding 4 p*K* units (see Table 4), to a lesser extent already visible in the log *P* predictions. Due to the directional nature of the distribution coefficient accounting for pH can only shift the partition coefficient to lower values because the ionized species is assumed to be unable to enter the organic phase. As a consequence, regression slopes deviate even more strongly from unity for log *D* compared to log *P* predictions, but the origin of the total error is probably related to both phases.

**Fig. 3:**
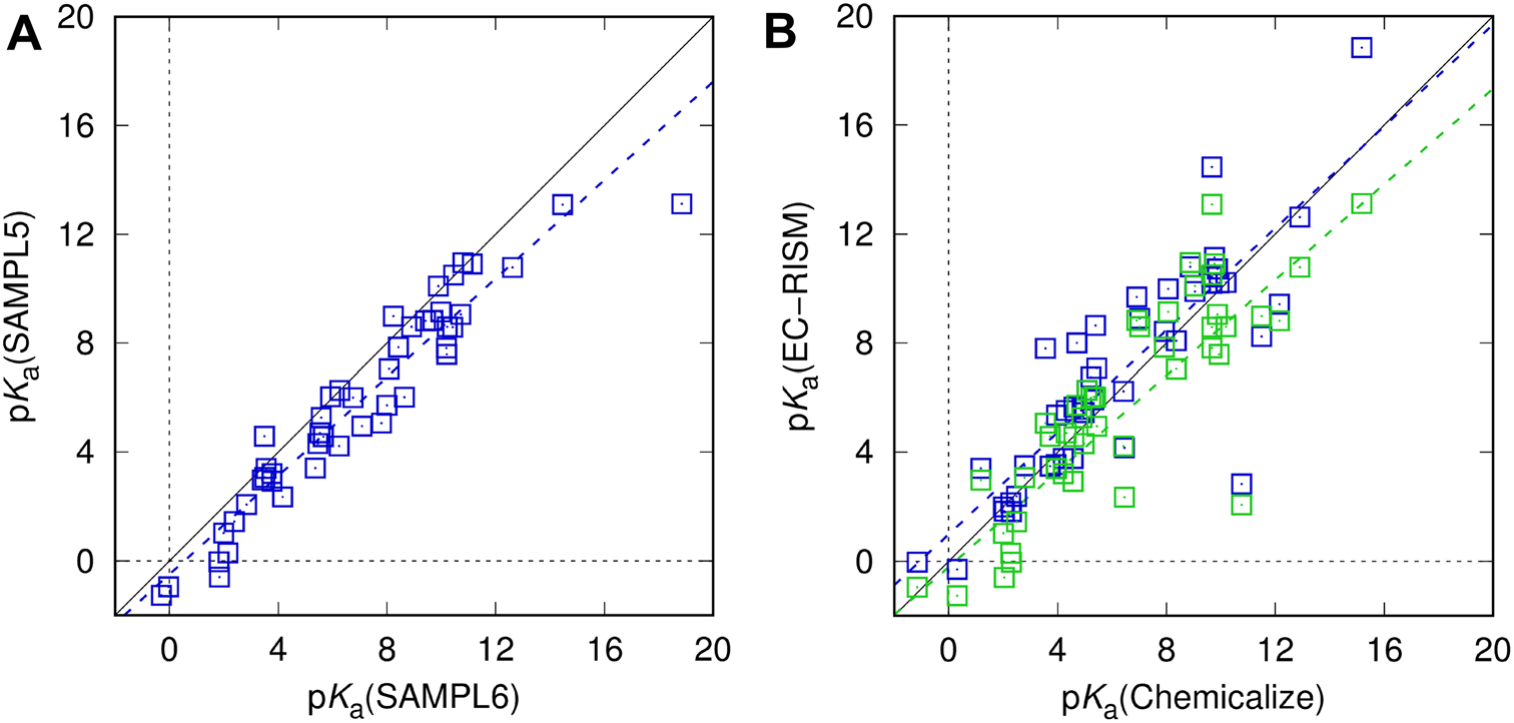
Acidity constants calculated with the original SAMPL5 setup compared to the SAMPL6 setup (**A**), results obtained from SAMPL5 (green) and SAMPL6 setups (dark blue) compared to Chemicalize [87] predictions (**B**). Dashed lines indicate descriptive regression results. Raw data are provided as Online Resource 5

Looking first at the effect of pH, we can test to what degree p*K*_a_ predictions change for the SAMPL5 compounds between SAMPL5 and SAMPL6 setups. Unfortunately, no experimental aqueous p*K*_a_ values are accessible for these compounds, but comparison of the p*K*_a_ values predicted using the SAMPL5 and SAMPL6 setup shows (Table 5; Fig. 3) that the predicted values of both setups have a good correlation, and those of the former are on average lower by about 1.15 p*K* units. Comparing these results with predictions from a different source, in this case using p*K*_a_ values empirically predicted using Chemicalize [87] shows that both methods have reasonable agreement with the empirical predictions with RMSEs of 2.10 and 2.07, respectively. The higher predicted p*K*_a_ values of the SAMPL6 setup lead to opposite effects for acids and bases. Acids will be predicted to have a lower fraction of the ionic species at pH 7.4 so their log *D* will be closer to the log *P*, while bases will be predicted to have a higher fraction of the ionic species and their log *D* will be shifted by a larger amount. Since there are 33 basic and only 14 acidic p*K*_a_ values this leads to a stronger effect of the p*K*_a_ on the already slightly lower partition coefficients predicted using the SAMPL6 setup when calculating the distribution coefficients, but the effect is not large enough to correct for the massive outliers near the negative limit of the dynamic range. Given the expected p*K*_a_ prediction uncertainty of the SAMPL6 setup of around 1 p*K* unit [69], it is very likely that p*K*_a_ errors can be ruled out as the source of the computational discrepancy.

**Fig. 4:**
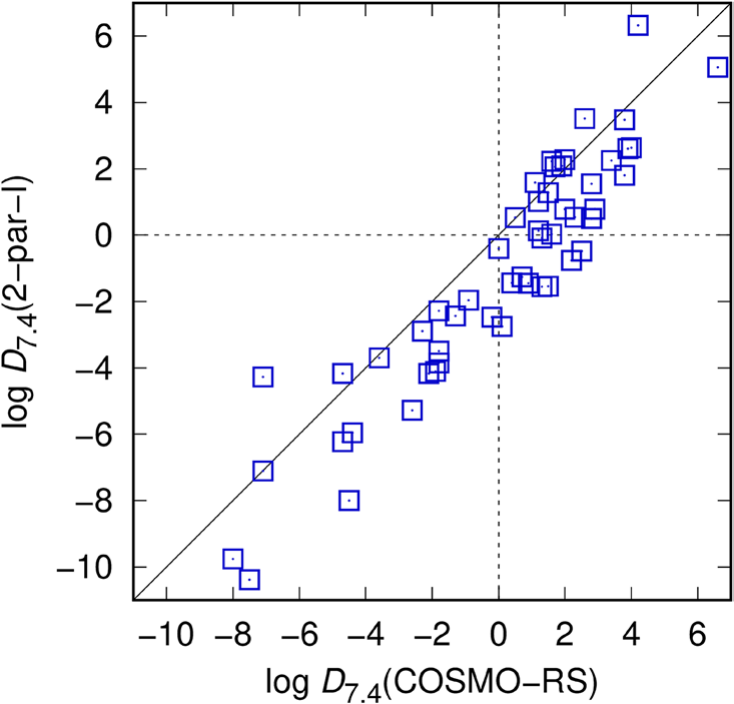
Distribution coefficients calculated using the best-performing SAMPL6-type EC-RISM model (2-par-I, RMSE 2.49, excluding SAMPL5_083) compared with predictions from the COSMO-RS model applied to the SAMPL5 challenge [89] with an RMSE of 2.11 (before correction for water presence, including special correction for SAMPL5_069). The diagonal line indicates perfect correlation

Model limitations attributed to the cyclohexane phase are another possible source of error. The EC-RISM models describe cyclohexane as a pure organic phase, ignoring a small, experimentally measurable water fraction estimated between 3.20 × 10^−4^ and 3.75 × 10^−4^ [88]. Especially for very polar compounds this might have a significant effect on the Gibbs energy of solvation in cyclohexane because single water molecules could reside near the polar solute over considerable time, as has been found during MD simulations by Bannan et al. [43]. These authors found a significant impact of added water molecules on the calculated log *P* for compound SAMPL5_074, changing from − 3.76 (no water) to − 2.82 (one water) and − 1.74 (7 water molecules), the last value being close to the experimental (log *D*) value of − 1.9. While this could explain the deviations to lower calculated distribution coefficients for the more polar compounds one should keep in mind that the water concentrations during these simulations were ~ 14–100 times higher than the experimental values. As the authors note, further investigations into the actual local water concentrations near the solute are necessary to understand the role of residual water in cyclohexane. Also, in the aftermath of the SAMPL5 challenge Klamt et al. studied the effect of small water concentrations and found only a minor improvement of some predicted values [89], yielding an RMSE of 2.08 (2.11 before accounting for the water fraction) from COSMO-RS calculations. Even when comparing the predicted distribution coefficients obtained by EC-RISM with their model that performed best in the original SAMPL5 challenge, including the correction for the water fraction, the agreement is significantly better than with the experimental data (Fig. 4). While there is an offset towards lower predicted values for EC-RISM which makes the performance slightly worse, there is a clear agreement between the two models even for the most hydrophilic and lipophilic compounds (RMSE of 1.77 relative to our best model 2-par-I). Hence, a systematic deficiency of the apolar phase model can neither be identified and, more importantly, two entirely *different prediction models* yield *similar disagreement* with experimental reference data with a relative RMSE that is even smaller than RMSEs with respect to experimental values in both cases.

The agreement between both approaches is even more evident if we analyze a reduced dataset excluding the seven worst outliers (implying the missing SAMPL5_083 as an effective outlier as well) similar to Klamt et al. [89] who excluded the eight worst outliers. In our case these are SAMPL5_033, 010, 015, 037, 063, 074, 081 (most of them predicted too small); the smallest deviation among these was found for SAMPL5_037 (3.77), the largest for 074 (7.86), all from applying the 2-par-I model. The resulting RMSE would drop to 1.37 (COSMO-RS: 1.57) with an MSE of only 0.12. Outliers near the limits of the dynamic range apparently account for the largest share of the discrepancies. All these findings point to a systematic problem either with the experiments or with the way theoretical models try to reflect experimental conditions, which will be further discussed in the concluding section.

### SAMPL2 revisited

**Fig. 5:**
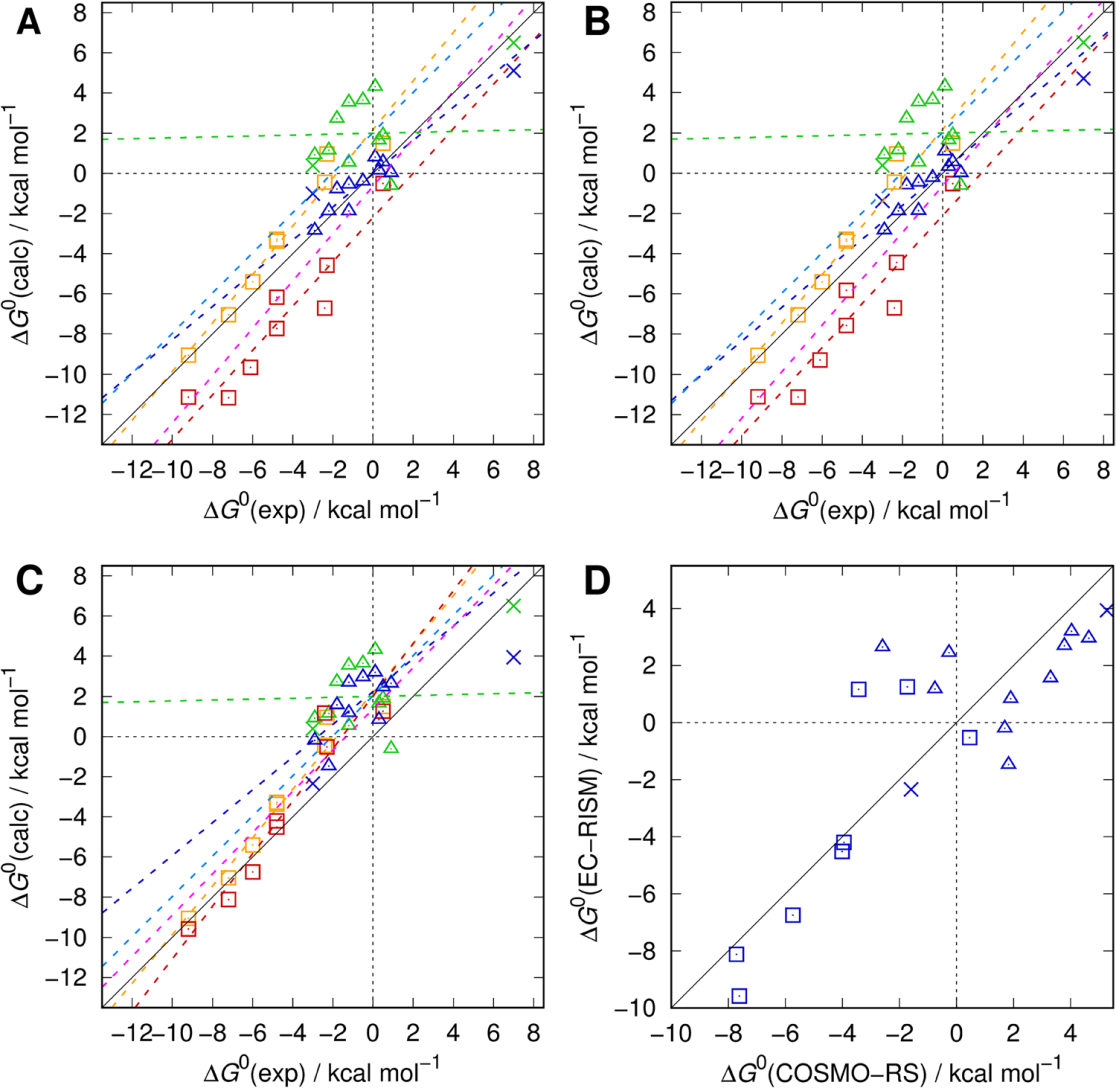
Calculated and experimental standard reaction Gibbs energies for the tautomer pairs of the SAMPL2 dataset (**A**–**C**) [37, 54] and comparison of explicit thermodynamic cycle data with corresponding explicit COSMO-RS (MP2+vib-CT-BP-TZVP) results [90] (**D**). Data using the SAMPL6 workflow (MP2/6-311+G(d,p)/*φ*_opt_/PSE-2) are shown as orange squares (obscure pairs 1-6), green triangles (explanatory pairs 10–16) and green crosses (explanatory pairs 7 and 8). Linear regressions are depicted as dashed lines in corresponding colors, with the total regression over all pairs in light blue (**A**–**C**). The data of the original SAMPL2 submission are shown by red squares (1–6), blue triangles (10–16) and blue crosses (7 and 8) with regression lines again in corresponding color and total regression in magenta for the best performing SAMPL2 model (MP2/aug-cc-pVDZ/PSE-3) using only minimum conformations for SAMPL2 setup (**A** SAMPL2/min and SAMPL6/*Z*) or the Boltzmann weighted free energies of the conformational ensemble (**B** SAMPL2/*Z* and SAMPL6/*Z*). Results from the explicit thermodynamic cycle combining SAMPL6-style Gibbs free energies of hydration and CCSD(T)/cc-pVTZ gas phase free energies including B3LYP/6-311+G(d,p) thermal corrections are shown by analogously color-coded symbols in (**C**)

Compared to the SAMPL5 re-analysis, the expectation was even higher to obtain improved results for the SAMPL2 tautomer datasets, as we are facing a simpler single-phase problem in this case, with results collected in Tables 6 and 7 and Fig. 5. During SAMPL2, training (“explanatory”) and test (“obscure”) sets were composed of different chemical and reaction classes, the former of keto-enol 5-membered heterocycles (compound numbers 10–16) and diketo compounds (7 and 8, both with considerably larger estimated experimental uncertainty and therefore excluded for training purposes by us earlier), the latter of keto-enol 6-membered heterocycles (1–6) [37, 54]. While we obtained very promising results during training with RMSEs of 0.58/0.66 kcal mol^−1^ for rotamer minima (“min”) or partition function (“*Z*”), respectively, corresponding metrics for the blind test set were much larger (RMSEs 2.90/2.78 kcal mol^−1^) yielding overall RMSEs of 1.98/1.93 kcal mol^−1^ which still was a major success back then. Moreover, we noted a systematic shift (measured by MSE and *b*′) for the 6-membered rings (keeping the keto-enol direction consistent as specified by the challenge organizers) which gave rise to the puzzling conclusion that the computational methodology was seemingly inconsistent depending on ring topology and composition.

**Table 6:** Statistical metrics (root-mean-square error RMSE/kcal mol^−1^, mean absolute error MAE/kcal mol^−1^, mean signed error MSE/kcal mol^−1^, and slope *m'*, intercept *b'*, and coefficient of determination *R*^2^ from descriptive regression) for all SAMPL2 tautomer pairs from SAMPL6-type models for water [MP2/6-311+G(d,p)/PSE-2] and the original SAMPL2 setup (MP2/aug-cc-pVDZ/PSE-3), the latter reported for calculations including minimum rotamer (“min”) free energies only and from partition function (*Z*) averaging, while SAMPL6-style calculations—also for explicit consideration of thermally corrected gas phase legs [“CCSD(T)”] of the thermodynamic cycle as in [71]—are shown for the partition function approach only

Model	Group	RMSE	MAE	MSE	*m*	*b*	*R*^2^
SAMPL6/*Z*							
1–6	Obscure	1.59	1.26	1.26	1.21	2.19	0.95
10–16	Explanatory	3.36	3.08	2.78	0.02	2.00	0.00
1–16	All	2.69	2.24	2.04	1.00	2.04	0.79
SAMPL6/CCSD(T)							
1–6	Obscure	1.52	1.13	0.62	1.31	2.02	0.93
10–16	Explanatory	2.62	2.39	2.39	0.82	2.24	0.46
1–16	All	2.20	1.83	1.32	1.03	1.38	0.79
SAMPL2/min							
1–6	Obscure	2.90 (2.91)	2.67	− 2.67	1.10	− 2.20	0.89
10–16	Explanatory	0.58 (0.57)	0.46	0.12	0.83 (0.89)	− 0.02 (− 0.05)	0.78 (0.77)
1–16	All	1.98 (1.93)	1.49	− 1.00	1.18	− 0.64 (− 0.63)	0.86
SAMPL2/*Z*							
1–6	Obscure	2.78	2.53	− 2.53	1.10	− 2.10	0.89
10–16	Explanatory	0.66	0.52	0.21	0.84	0.09	0.74
1–16	All	1.93	1.47	− 0.94	1.16	− 0.63	0.86

Numbers in parentheses denote original values from the SAMPL2 paper [54] where equilibrium constants have been transformed to reaction Gibbs energies, whereas we here show metrics relative to reference Gibbs energies from the SAMPL2 overview paper [37]. Structures are provided as Online Resource 6; calculated data, also split into separate components, as Online Resource 7

**Table 7:** Experimental [37] tautomerization Gibbs energies (kcal mol^−1^) including estimated errors, calculated values from original SAMPL2 setup for rotamer minima (“min”) and partition functions (“*Z*”) [54], and from direct (“*Z*”) and indirect (“CCSD(T)”) [71] approaches using the SAMPL6 setup

Reaction	Exp	Error	SAMPL2/min	SAMPL2/*Z*	SAMPL6/*Z*	SAMPL6/CCSD(T)
1A → 1B	− 4.8	0.3	− 7.73	− 7.57	− 3.38	− 4.52
2A → 2B	− 6.1	0.3	− 9.66	− 9.29	− 5.40	− 6.74
3A → 3B	− 7.2	0.3	− 11.17	− 11.12	− 7.04	− 8.12
4A → 4B	− 2.3	0.4	− 4.57	− 4.43	0.96	− 0.52
5A → 5B	− 4.8	0.5	− 6.16	− 5.83	− 3.28	− 4.19
5B → 5C	0.5	0.2	− 0.51	− 0.51	1.50	1.25
6A → 6B	− 9.2	0.4	− 11.15	− 11.12	− 9.05	− 9.59
6A → 6Z	− 2.4	0.3	− 6.72	− 6.69	− 0.43	1.17
7A → 7B	7.0	1.5	5.11	4.71	6.50	3.94
8A → 8B	− 3.0	3.0	− 1.01	− 1.38	0.38	− 2.34
10B → 10C	− 2.9	0.4	− 2.84	− 2.83	0.91	− 0.20
10D → 10C	− 1.2	0.2	− 0.55	− 0.45	3.54	2.70
11D → 11C	− 0.5	0.2	− 0.39	− 0.23	3.64	2.96
12D → 12C	− 1.8	0.7	− 0.79	− 0.60	2.73	1.57
13D → 13C	0.1	0.1	0.81	1.09	4.31	3.20
13D → 14C	0.3	0.3	0.16	0.32	1.64	0.84
15A → 15B	0.9	0.3	0.02	0.01	− 0.62	2.65
15A → 15C	− 1.2	0.3	− 1.87	− 1.87	0.53	1.18
15B → 15C	− 2.2	0.3	− 1.88	− 1.88	1.15	− 1.47
16A → 16C	0.5	0.1	0.56	0.56	1.90	2.46

The results from applying the direct and the indirect SAMPL6 setups [71] did, much to our surprise, not settle the inconsistency. While the test set error decreased considerably to ca. 1.5 kcal mol^−1^, the training set performance deteriorated down to an RMSE of 2.6–3.4 kcal mol^−1^, with the smaller number obtained by the expectedly more reliable explicit CCSD(T) gas phase approach. Total RMSEs averaged over training and test set are finally even worse (2.2–2.7 kcal mol^−1^), again with a better performing explicit CCSD(T) model. Taking all metrics together, the explicit gas phase thermodynamic cycle approach performs best and most consistent among all compound classes, but the performance inversion compared to the earlier SAMPL2 results is worrisome. Equally worrying is the finding that more advanced methods apparently do not improve predictive power overall, though we were able to produce better balanced results by refining computational methods. Whether or not there exists an experimental problem with the training compounds 10–16 remains elusive at this point. One hint may be that the partition function approach in SAMPL2 produced slightly worse results, quite in contrast to compounds 1–6.

As for the SAMPL5 re-analysis, more insight can be gained from comparison with results from technically very different, though still QM-based models, here again by relating our results to COSMO-RS data. In the aftermath to the SAMPL2 challenge, Klamt and Diedenhofen [90] presented an enhancement over their original submission. Compared to us, they obtained an inverse trend, worse performance for the training compared to the test set, and they augmented hydration free energies with explicit gas phase calculations (MP2+vib-CT-BP-TZVP), similar to our SAMPL6/CCSD(T) approach. The corresponding juxtaposition is shown in Fig. 5D. The similarity between the two approaches particularly for the strongly negative values is striking while the 5-membered ring data distribution scatters more strongly (RMSEs with respect to experiment of 2.62/3.82 for 10–16 and 1.52/1.50 kcal mol^−1^ for 1–6, comparing SAMPL6/CCSD(T) and MP2+vib-CT-BP-TZVP, respectively, see also Online Resource 6). This provides strong evidence that experimental reference data for the “obscure” test set are reliable whereas the “explanatory” training set raises some doubts, despite the estimated small experimental uncertainties published. Moreover, by averaging over both methods, a hypothetical consensus prediction is obtained, for which the RMSEs relative to both original predictions are smaller than each individual prediction with respect to experiment, dropping to only 1.07 (1–6), 1.25 (10–16), and 1.12 (1–16) kcal mol^−1^. This computational consistency, particularly for the crucial pairs 10–16 whose original RMSEs were more than twice as large, together with the individual divergence from experiment suggests that experimental values for the explanatory set pairs 10–16 should be reconsidered.

## Concluding discussion

What did we learn over the past decade? The common key result, observed as average over re-analysis of all SAMPL2 and SAMPL5 datasets is—at first sight—that we did not make any visible progress, with a persisting log *D* or free energy uncertainty of around 2 p*K* units or 2 kcal mol^−1^, respectively. At second sight, the situation is, however, much more complicated and provides essential insight into computational and experimental pitfalls.

The “vertical” way, comparing methods with advancing performance over time on the same original dataset and a “horizontal” approach, extending the size or diversity of the data source while employing one and the same method, reveal different aspects of model performance. In the latter case, only the bias originating from training or calibrating models with limited datasets can be elucidated, not the quality of the data or the models themselves. In contrast, the vertical approach utilized in this work provides insight into expectation bias which can be the related to both, computational and experimental issues to be analyzed further. Moreover, augmentation of the vertical approach by direct comparison with other challenge participants as done here, which is only possible by prediction challenges stimulating participation of a large number of groups, can be useful for discriminating between experimental and modeling problems, or both.

Coming back to the results obtained in the present work, the major surprise came with the disappointing insight that derived data from independently optimized computational methodology did not correspond to advanced predictive power. We have indeed succeeded over the past years to bring the error of direct application of 3D RISM theory to thermodynamic problems down to an order of 1 p*K* unit or 1–2 kcal mol^−1^ for solvation free energies. Another layer of calibration made it possible to even predict acidity constants to within a similar accuracy. Yet, for composite problems such as a distribution coefficient in SAMPL5, which can be physically and exactly traced back to solvation free energy and acidity calculations, the accuracy deteriorates (although the partition coefficient calculation was quite reliable during SAMPL6 part II, but that might be related to the small dynamic range). Hence, our “conservative” approach to model basic physical quantities only and compute derived data by exact thermodynamics could potentially suffer from non-canceling or even amplified errors.

Leaving the obvious alternative possibility of erroneous experiments aside, such as a low equilibration time and the possibility of detector saturation [91, 92], the key questions therefore are: Do theoretical models really mimic the experimental reality? And what can be done within both domains to converge to a common well-defined reality that allows for truly unbiased assessments of model performance? At least for the log *D* problem there are indeed issues that are typically ignored or underestimated. Theoretically, we are essentially doing the right thing when we try to compute a thermodynamic standard quantity (as is required for the strict definition of equilibrium constants like log *P* or log *D*) by referencing to the infinite dilution limit and treating all non-ideal mixture effects (even formally including phenomena such as aggregation) via appropriately chosen activity coefficients. In the absence of a predictive activity coefficient model for diverse compounds in various solvents, this would in turn demand that experiments adequately extrapolate to the infinite dilution case, which is not easily guaranteed. Indeed, the accumulation of log *D* outliers near the extremes of the dynamic range (i.e. high solubility in either water or cyclohexane) found by us and by others, hint at an experimental problem. Hill and Young reported a general issue with the computational prediction of distribution coefficients caused by low solubilities of very hydrophilic and very lipophilic compounds in the organic and the aqueous phase, respectively [93] (though specifically for octanol–water, but probably transferable to other nonaqueous solvents). As near the extremes we will always observe a combination of low solubility in one phase with high solubility in the other, measurement uncertainty can affect the low-solubility side whereas non-unity activity coefficients can be relevant for the high-solubility regime. These are therefore the urgent questions for the next phase of experimental–computational co-design.

As we did on the computational side, repeating experiments in a vertical way, i.e. following after some time using possibly enhanced experimental equipment or protocols, should also become common practice. This is particularly challenging for the problem of tautomer determination in an aqueous environment which is generally known to be problematic, as also indicated by our consensus estimate over different computational models that is more consistent internally than with experimental reference data in the SAMPL2 case. Proper enumeration and fast population predictions are of utmost importance for future model improvements, as the combinatorial problem grows dramatically with the number of protonatable groups. In practice, this will ultimately require sampling protocols like those used in constant-pH simulations that require accurate (de-)protonation free energies for estimating state switching probabilities. Uncertainties in this respect revealed by the SAMPL2 re-analysis could therefore have massive impact on derived quantities when proton shifts play a role. Our data clearly indicate that outliers identified from consensus correlation analysis should stimulate re-assessment on the experimental side.

On the other hand, the fact that not only experiments are a source of uncertainty is clearly revealed by repeating the SAMPL2 workflow using presumably better methodology. The inconsistency between direct and indirect approaches obviously demonstrates room for improvement, in our case very likely by adjusting nonbonded dispersion–repulsion parameters and re-addressing the corrective schemes toward more accurate chemical excess potentials. Reaching consistency between different methodologies can be a useful way to optimize computational strategies even in the absence of reliable experimental data.

Existing knowledge on the respective accuracy is essential for industrial applications. Our current results clearly show that thorough benchmarking of methods is warranted to assess their accuracy in order to choose those suited best for the scientific question at hand and avoid repeating method evaluations. However, successful benchmarking can only be achieved if it is based on high-quality data. In this context, quality of data not only means measurement precision, but has also to be viewed in light of dataset size, structural diversity, and sustainable, reproducible measurement protocols. Traditionally, this is the domain of industry where data is acquired in a consistent manner over long periods of time. In light of current trends toward “open data”, efficient research data management, the FAIR principles, and the relevance of reliable experimental and computational data for developing powerful machine learning models, this constitutes a common goal for industry and academia working together.

## Electronic supplementary material

Below is the link to the electronic supplementary material.

Supplementary file1 (GZ 65 kb)

Supplementary file2 (GZ 12 kb)

Supplementary file3 (GZ 871 kb)

Supplementary file4 (GZ 32 kb)

Supplementary file5 (GZ 15 kb)

Supplementary file6 (GZ 68 kb)

Supplementary file7 (GZ 7 kb)

Supplementary file8 (TXT 2 kb)
